# Integrating HIV/AIDS services into financial protection systems to increase sustainability of the HIV/AIDS response

**DOI:** 10.1186/s12913-025-12528-9

**Published:** 2025-05-28

**Authors:** Judy Chang, Mai Hijazi, Susanna Baker, Onyeka Igboelina, Carlyn Mann, Hannah Marqusee, Cam Anh Nguyen, Carolina Piña, Jemeh Pius, Robert Stanley, Zacheaus Zeh Akiy

**Affiliations:** 1 Office of HIV/AIDS, Global Health Bureau, U.S. Agency for International Development (USAID), Washington DC, USA; 2 HIV/AIDS and Tuberculosis Office, USAID Nigeria, Abuja, Nigeria; 3 Office of Development Cooperation; Policy, Planning, and Learning Bureau; USAID, Washington DC, USA; 4 Office of Public Health and Education, USAID Cambodia, Phnom Penh, Cambodia; 5 Health Office, USAID Vietnam, Hanoi, Vietnam; 6 Health Office, USAID Dominican Republic, Santo Domingo, Dominican Republic; 7 Health Office, USAID Tanzania, Dar es Salaam, Tanzania; 8 Health Office, USAID Cameroon, Yaoundé, Cameroon

**Keywords:** Financial protection, Health financing, Health insurance, Health systems strengthening, HIV/AIDS, Sustainability, Universal health coverage

## Abstract

Begun in 2014, the Sustainable Financing Initiative (SFI) was dedicated to mobilizing domestic resources for the HIV response. Among its three programmatic pillars was a focus on ensuring financial protection for people living with HIV (PLHIV). SFI’s activities were founded on a strong understanding of and alignment with partner government priorities, as well as costing and actuarial analyses, which allowed for the development of context-specific approaches for improving financial protection for PLHIV. SFI implemented financial protection activities in a total of nine countries; the five countries with the most substantial investments are discussed in this paper. In Vietnam, SFI’s support helped the country to integrate almost all outpatient HIV treatment facilities into the public health system, attain enrollment of 90% of PLHIV into Social Health Insurance (SHI), and increase domestic funding for HIV through SHI. In Cambodia, SFI supported the development of a guiding framework for integration of HIV/AIDS services into the existing health system; key achievements included a policy change that expanded eligibility for the country’s Health Equity Fund, allowing all PLHIV to access free health services. In the Dominican Republic, SFI support led to the inclusion of antiretroviral drugs in the family health insurance benefit package, increased enrollment of PLHIV in health insurance, and expanded care options through non-governmental organizations. In Nigeria, SFI’s support helped to enroll more than 600,000 people and empanel 216 health facilities into the Lagos State Health Insurance Scheme. In Cameroon, SFI support helped the government achieve stepwise progress on key building blocks of its planned new universal health coverage system; SFI contributed to the development of a consolidated package of services, standardized care and service protocols, and accreditation criteria. SFI’s investments in financial protection demonstrated that with strong political will; long-term engagement with partner governments; and focused technical assistance for advocacy, policy reform, and implementation support, HIV services can be successfully integrated into financial protection systems. Such integration can promote increased, long-term domestic financing for HIV while also protecting PLHIV from financial risk.

## Background

Over the past two decades, high levels of donor assistance for HIV have helped to expand access to HIV prevention and treatment services across low- and middle-income countries. Donors such as the U.S. President’s Emergency Plan for AIDS Relief (PEPFAR) and the Global Fund to Fight AIDS, Tuberculosis and Malaria (GF) have placed a focus on equitable access to services for vulnerable and at-risk populations, and evidence shows that these programs have largely been pro-poor [1]. In addition, donors have also helped to drive policy changes that increase access to services. For example, over the past several years, PEPFAR has worked with governments to abolish user fees for HIV services in the public sector, helping to ensure that direct out-of-pocket costs do not pose a financial barrier to accessing services at the point of care.

Although donors still fund a significant proportion of the HIV response in low- and middle-income countries (37%, or $8.2 billion, in 2020) [2], there is a long-term trend of decreasing donor funding for HIV [3]. Over the past decade, bilateral donor funding for HIV, excluding the U.S. contribution, has declined by more than $1 billion [2]. U.S. government funding for HIV, largely flatlined over the last several years, is also expected to decline in coming years. Furthermore, the longer-term global impact of the COVID-19 pandemic has the potential to significantly shift donor funding priorities moving forward. As donor funding declines, it is imperative that we have effective strategies in place to sustain domestic financing for high-quality HIV services while also ensuring financial protection for people living with HIV (PLHIV). According to the World Health Organization, “financial protection is achieved when direct payments made to obtain health services do not expose people to financial hardship and do not threaten living standards” [4].

One strategy for sustaining financing for HIV is to integrate HIV into existing or developing financial protection systems. Globally, a number of upper-middle-income countries–both in high-prevalence countries with generalized HIV epidemics and in lower-prevalence countries with concentrated epidemics–have made progress in integrating HIV into national health insurance schemes. For example, Thailand was a leader in integrating HIV into its Universal Coverage Scheme, with antiretroviral drugs (ARVs) fully integrated by 2006 [5]. South Africa’s 2017 National Health Insurance Policy, which aims to provide equitable and adequate financial risk protection for all citizens, also includes HIV [6]. However, in most low-income and lower-middle-income countries, incorporating HIV had largely been ignored to date because of the high levels of donor dependence.

While there is strong evidence of the benefits of integrating HIV and other health services for both HIV and non-HIV health outcomes [7], there has been less systematic documentation of the effects of integrating HIV into financial protection systems. Nevertheless, targeting financial protection systems for integration has been seen as an opportunity for broader systems integration and sustainability [8], with plausible benefits ranging from more effective risk pooling and reduced costs associated with parallel HIV systems to increased domestic resource allocation for HIV and better access to care for clients. Moreover, given that donor funding for HIV has helped to limit out-of-pocket spending for HIV [9], such integration can also play an important role in protecting PLHIV from increasing out-of-pocket costs as financing shifts from donors to domestic sources. These efforts can be further facilitated by aligning with the current global movement towards universal health coverage (UHC) and achievement of Sustainable Development Sub-goal 3.8 [10].

This paper focuses on efforts to improve financial protection for PLHIV under the Sustainable Financing Initiative (SFI). Led by USAID from 2014 to 2022, SFI was a $48 million PEPFAR-funded initiative dedicated to mobilizing domestic resources for the HIV response [11]. SFI was organized around three programmatic pillars: ensuring financial protection of PLHIV, engaging the private sector [12], and improving public financial management [13]. This paper outlines the approach and summarizes key results and lessons learned from SFI’s implementation of financial protection activities in five countries and offers recommendations for the next generation of programs focused on financial protection for HIV. SFI’s financial protection activities focused on various country-specific health insurance and social protection schemes funded by a combination of individual contributions, taxes, and/or donor funding, rather than solely contribution-based schemes. The specific terminology used in this manuscript for each of these schemes reflects the terminology used by the country.

## Program approach

At the onset of the HIV epidemic and PEPFAR’s launch in 2003, HIV services were initiated as an emergency response, often through programs operating in parallel to countries’ existing primary health care systems. Over the decades since, HIV programs have matured, with 34% of PEPFAR-supported countries having attained epidemic control by 2020 [14]. As they reach this point, many countries are looking to integrate HIV into their broader health systems, both from a service delivery and a financing perspective.

SFI’s financial protection activities were designed to achieve a two-fold benefit. By including HIV in financial protection schemes and supporting PLHIV enrollment, PLHIV would have increased access to both HIV and non-HIV services, while also protecting them from potential increases in out-of-pocket costs as donor funding for HIV declines. Furthermore, integrating HIV into financial protection systems would reduce parallel systems, increase government ownership, and ensure that HIV was included in the financing of the overall health system. Assuming increasing government financial commitment to the health system, such integration would increase the long-term financial sustainability of the HIV response. Given the myriad potential challenges associated with implementing financial protection systems in low- and middle-income countries, SFI activities incorporated targeted technical assistance to help address key risks in each country.

In the early planning phases, SFI recognized that integration of HIV into financial protection systems would require significant political will, an appropriate level of financial readiness, mature health systems, and a tailored approach specific to each individual country’s context. Countries were therefore selected based on an assessment of: (1) host government commitment, including demonstrated leadership and willingness to make necessary policy changes; (2) host government financial readiness, including established financial systems and evidence of political will to start increasing government financial contributions to health and the HIV response; (3) country epidemic and response context; and (4) USAID staffing in country, recognizing that ongoing engagement with host government counterparts would be essential for successful implementation.

Once countries were selected, SFI took a methodical, evidence-based approach to introduce its interventions. Activities were founded on an understanding of and alignment with host government priorities. In addition, costing and actuarial analyses were used to build an analytical base for all activities. These analyses considered the nature of the epidemic and total burden of HIV, given the impact these factors have on overall resource requirements. This foundation was pivotal for effectively advocating for and building political will to support SFI’s activities and the broader systems reforms required to achieve our joint objectives.

As interventions were planned, SFI adapted approaches to individual country contexts. For example, in some countries such as Vietnam and Cambodia, HIV was added to existing financial protection systems; in others such as Cameroon and Nigeria, SFI provided support as the financial protection systems were built, ensuring that HIV was incorporated from the start. Systems adjustments were also required, in some cases integrating PEPFAR’s parallel systems within broader country systems.

## Results

SFI implemented financial protection activities in a total of nine countries. The five countries with the most substantial investments–Vietnam, Cambodia, Dominican Republic (DR), Nigeria, and Cameroon–are discussed in the following sections of this paper. In the other four countries–Botswana, Côte d’Ivoire, Namibia, and Tanzania–SFI supported mostly analytical work. In Namibia and Botswana, SFI supported the respective partner governments in addressing inefficiencies within their medical aid schemes. In Botswana, SFI achieved this by developing a cost recovery implementation plan and tariff setting roadmap, while in Namibia, SFI conducted cost analyses and developed an implementation guide for a more cost-effective ARV procurement mechanism. In Côte d’Ivoire, SFI carried out analyses of user fees charged for HIV services and the financial implications of eliminating HIV user fees for the health system. In Tanzania, SFI supported a six-month pilot to provide low-cost health insurance through an existing mobile-enabled private microinsurance product.

Table 1 details the HIV landscape, and Table 2 the health financing landscape, of the five focus countries at baseline (2014 or closest available data) and endline (most recent available data). In the following sections, we detail SFI’s work in each of these countries, providing an overview of key contextual factors, activities implemented, results, and future outlook and opportunities. The activities and results are also summarized in Table 3. In some countries, additional PEPFAR funding was secured to build on SFI investments, and the results of these joint investments are presented here.

**Table 1:** HIV landscape of the five SFI financial protection focus countries [15]

Country	HIV prevalence among 15–49 year olds		Total number of PLHIV		95-95-95 status					
					% of PLHIV diagnosed		% of PLHIV diagnosed who are on treatment		% of PLHIV on treatment who are virally suppressed	
	2014	2020	2014	2020	2014	2020	2014	2020	2014	2020
**Vietnam**	0.4%	0.3%	230,000	250,000	Data not available	86% (2021) [16]	Data not available	83% (2021) [16]	Data not available	96% (2021) [16]
**Cambodia**	0.7%	0.5%	80,000	75,000	68%	84%	94%	> 98%	Data not available	97%
**Dominican Republic**	1.0%	0.9%	72,000	75,000	50%	82%	66%	62%	Data not available	96%
**Nigeria**	1.4%	1.3%	1,500,000	1,700,000	58%	90%	79%	96%	Data not available	84%
**Cameroon**	3.8%	3.0%	520,000	500,000	59%	78%	47%	95%	Data not available	Data not available

**Table 2:** Health financing landscape of the five SFI financial protection focus countries

Country	Current health expenditure (CHE) per capita (US$) [17]		Development assistance for health as % of CHE [18]		Out-of-pocket spending as % of CHE [17]		% of HIV expenditures partner government funded		Economic classification (2021) [19]	Status of financial protection systems at outset of SFI interventions
	2014	2019	2014	2019	2014	2019				
**Vietnam**	$118	$174	3%	1%	42%	45%	Data not available	49% (2020) [20]	Lower- middle income	Social health insurance in place with substantial population coverage but limited coverage of HIV services
**Cambodia**	$74	$115	14%	7%	59%	64%	14% (2014) [18]	24% (2017) [18]	Lower- middle income	Social protection scheme in place, but only poor PLHIV eligible
**Dominican Republic**	$313	$347	2%	1%	31%	28%	33% (2014) [21]	61% (2019) [20]	Upper- middle income	Social health insurance in place, but ARVs not covered and limited enrollment of PLHIV
**Nigeria**	$106	$70	8%	7%	72%	71%	15% (2015) [21]	17% (2021) [21]	Lower- middle income	National health insurance scheme in place, but very low enrollment focused on the formal sector
**Cameroon**	$62	$56	9%	13%	65%	72%	Data not available	13% (2017) [22]	Lower- middle income	Initial stages of establishing UHC scheme

**Table 3:** Summary of activities and results from SFI’s financial protection activities in five countries

Country/Timeline	Activities implemented	Key results
**Vietnam** 2015-present	- Cost analyses and actuarial analyses - Strengthening of policy and legal frameworks - Technical assistance for integration of HIV services and procurement of ARVs through SHI - Implementation assistance to health facilities to operationalize SHI - Outreach to PLHIV to facilitate enrollment into SHI - Advocacy for local government contributions to SHI premiums and co-payments for vulnerable populations	- 90% of PLHIV enrolled in SHI - 98% (438 out of 446) of outpatient treatment facilities integrated into SHI and receiving reimbursements - 48 out of Vietnam’s 63 provinces committed $1,122,000 for client-level ARV co-payments (2019–2020) - Domestic financing for HIV has grown, from funding 34% of the HIV response in 2015 to 53% in 2020 - $2.25 leveraged for every $1 of PEPFAR funds invested (through 2020)
**Cambodia** 2017-present	- Joint sustainability planning with the RGC and other stakeholders - Development of a framework for integrating HIV services into the public health system - Evidence generation and advocacy for increased domestic HIV funding, including to meet GF co-financing requirements - Advocacy to allow all PLHIV to access the HEF	- Fifth National Strategic Plan included financing for HIV services, committing the RGC to fund 50% of the HIV response by 2023 - Policy change enacted to allow 100% of PLHIV to access free healthcare services through the HEF, up from 38% of PLHIV in 2018 - Government committed $16.5 million for ARV procurement from 2018–2023 and $5 million per year thereafter
**Dominican Republic** 2017–2021	- Estimation of health insurance financing requirements and support for health insurance policy reforms - Technical assistance to integrate ARV financing and procurement into SENASA - Support for NGO participation in the national health insurance scheme - Strengthening of health worker capacity to increase health insurance enrollment for PLHIV	- Identified efficiencies in ARV warehousing and distribution projected to save the government an estimated $160,000 annually - 6,700 PLHIV newly enrolled in SENASA’s subsidized health insurance scheme (as of December 2020) - Three NGOs contracted with SENASA and have received $111,300 in reimbursements (as of May 2021)
**Nigeria** 2016-present	- Cost and actuarial analyses - Building on UHC as a political priority and engaging legislators as advocates - Advocacy for inclusion of HIV in national health insurance guidelines - Implementation support, including roadmap development and enrollment support, for the Lagos State Health Scheme	- Created a roadmap, endorsed by the national health insurance scheme, which states can follow to integrate HIV into their insurance schemes - 216 health facilities empanelled in the Lagos State Health Scheme; 197 of the empanelled facilities are providing and receiving reimbursements for HIV testing services (as of May 2022) - 611,578 people enrolled in the Lagos State Health Scheme (as of May 2022), compared to baseline of 11,000 (September 2020) - New National Domestic Resource Mobilization and Sustainability Strategy for HIV launched for 2021–2025
**Cameroon** 2018-present	- Technical assistance for the development of a UHC investment case - Training and capacity building of Ministry of Public Health staff on costing and budgeting tools - Support for development of a consolidated package of services, standardized care and service protocols, and health facility accreditation tools - Development of strategic communications materials and trainings to support UHC rollout	- Finalized consolidated package of interventions, including HIV services, to be included in UHC - Consolidated package of interventions costed - UHC communications strategy, plan, and tools developed - Health facility UHC accreditation tools developed and validated

### Vietnam

#### Context

Vietnam has a concentrated HIV epidemic with current prevalence at 0.3% and an estimated 250,000 PLHIV [15]. Expanding on earlier national health insurance schemes, Vietnam approved a plan for UHC in 2012, with the aim of expanding coverage to 80% of the population by 2020 and reducing client out-of-pocket costs to less than 40% of total health care spending [23]. The Social Health Insurance (SHI) scheme achieved 80% coverage by 2017, and 100% coverage was established as a subsequent target for 2020 [23]. Although SHI was meant to include HIV services, extensive donor funding from PEPFAR and GF continued to heavily subsidize Vietnam’s HIV response. Nevertheless, the already well-developed SHI provided a strong platform to which HIV could be added.

#### Activities

Vietnam’s graduation to middle-income country status and continued progress with management of the HIV response enabled the transition of greater financing and management responsibilities to the Government of Vietnam (GVN). SFI provided technical assistance for the transition process over several years, working closely with the Ministry of Health, the Vietnam Administration for AIDS Control (VAAC), and the Vietnam Social Security Agency to develop plans for integration of services, procurement of ARVs, and addressing long-term sustainability of the HIV program through the national SHI scheme.

Specifically, SFI developed evidence, including cost and actuarial analyses, to advance implementation of GVN’s policy for SHI as the primary financing mechanism for HIV services. SFI also directly supported management and administrative reforms to integrate donor-supported outpatient HIV treatment facilities into the public health system, driving efficiencies to reduce costs with the shift to GVN-managed programs. In collaboration with the VAAC and the Vietnam Social Security Agency, a stepwise programmatic and legal approach was developed to initiate transition of donor-funded HIV outpatient care clinics into ones that are eligible for SHI, and to enable clinic reimbursement for HIV-related services. SFI developed a liability model, which estimated the total costs of treatment for HIV, broken down by payer; this analysis demonstrated that the SHI fund could absorb the projected costs of HIV services.

SFI also provided assistance to set up a central procurement unit for managing HIV commodities and to establish competitive bidding processes for the procurement of ARVs [23]. Significant efforts were dedicated to increasing PLHIV enrollment under SHI through outreach, support with insurance card applications, and subsidization of co-payments for eligible clients. Through financial forecasting and targeted advocacy, SFI assisted the annual budget planning process for provincial level subsidies to support the ARV co-payments required by SHI. SFI also placed a strong focus on strengthening the capability and readiness of clinics, the health system, and SHI to successfully manage the transition and absorb services.

#### Results

From 2015 to 2020, SHI implementation for HIV treatment services scaled rapidly. By 2019, 90% of PLHIV were enrolled in SHI [24], up from approximately 40% at baseline [23]. By April 2020, 98% of the country’s HIV treatment facilities had been integrated into SHI and were receiving SHI reimbursements [24]. SHI began reimbursement of ARVs in March 2019, and by 2021, 82,000 PLHIV had received SHI-covered ARVs and HIV services, with ARV funding estimated at $11 million [25]. By 2020, 48 out of the 63 provinces had also committed $1,122,000 for client-level ARV co-payments and are renewing their financial commitment for the next five-year period of 2021 to 2025 [24]. The program saw a successful transition to fixed-dose combination tenofovir, lamivudine, and dolutegravir as the preferred ARV drug in 2021, with procurement planned to cover 103,000 clients in 2022 and up to 130,000 clients in 2023 [26].

The return on investment for SFI’s work to support the transition of HIV service coverage to the GVN’s SHI scheme is estimated to be $2.22 leveraged for every $1 invested by PEPFAR. Returns were quantified as the amount of financial resources mobilized, or saved, through: (1) the total SHI contribution value for HIV services reimbursed by the GVN in 2019; (2) the total budget allocated by provinces in 2019 to subsidize SHI premiums and co-payments; (3) estimated national SHI savings as a result of multi-month dispensing of ARVs; and (4) additional government budget execution for ARV procurement (2015–2016) and additional SHI commitment for ARV procurement (March 2019-December 2020).

#### Outlook

Vietnam has made considerable progress towards epidemic control while also taking on greater financial leadership for its HIV response. National budget allocations for HIV have grown significantly, with the proportion of the HIV response funded by domestic resources increasing from 34% in 2015 to an estimated 53% in 2020 [27]. The GVN’s SHI contributions have increased significantly since it started procuring ARVs for SHI in 2019, and its current goal is to cover 82% of all ARV needs by 2023 [26]. Provincial governments are also expanding financing plans for HIV, including expanding social contracting with community or private sector organizations for HIV prevention services. However, there are still ongoing SHI implementation challenges, including ensuring timely site reimbursement for services, continued support for ARV co-payment subsidies, and inclusion of newly recommended superior regimen ARVs within the SHI benefits package. Moreover, as it was originally designed as a curative scheme, the SHI benefit package does not include any prevention services, let alone HIV prevention services; in order to expand the SHI benefit package to include prevention services in the future, critical revisions to the current SHI Law and SHI’s Basic Health Service Package will be required.

### Cambodia

#### Context

Cambodia was one of the first countries in Asia to reach the UNAIDS 90-90-90 targets [28]. Successful programs funded and supported through PEPFAR and GF, strong commitment from the Royal Government of Cambodia (RGC), and active local civil society organizations were critical for achieving this significant milestone in 2017. This success, steadily decreasing donor funding, and Cambodia’s designation as a lower-middle-income country in 2015 brought to the forefront an urgent need to plan for the financial sustainability of the country’s HIV response. Over the last few years, Cambodia has worked to ensure the financial protection of PLHIV, both by increasing its financial commitments to the HIV response and through the country’s Health Equity Fund (HEF). The HEF is a social health protection scheme which provides free healthcare for the poor and vulnerable, reaching approximately three million Cambodians as of 2017 [29]. RGC funding of the HEF increased from 61% in 2017 to 71% in 2019, with an objective of reaching 100% domestic funding by 2025 [29].

#### Activities

Working with Cambodia’s Ministry of Health, the National AIDS Authority (NAA), the National Center for HIV/AIDS Dermatology and STD, UNAIDS, and the GF, SFI supported a coordinated sustainability planning process. This process included the development of a guiding framework for integration of donor-funded, vertical HIV services into the existing health system; inclusion of HIV financing goals in the national strategic plan; and advocacy for increased GF co-funding requirements.

In addition, SFI advocated for the expansion of the HEF to include all PLHIV, and for the HEF to incentivize increased HIV service provision by providing additional funding to health facilities. To support this advocacy, SFI developed cost estimates for scaling up coverage and supported the NAA to complete a vulnerability analysis of PLHIV.

#### Results

In 2018, SFI—in coordination with UNAIDS, the NAA, and development partners—supported the development of a transition readiness assessment and sustainability roadmap to identify key risks to a sustainable HIV/AIDS response. The roadmap, which covered service delivery, the critical role of civil society organizations in the HIV response, and financing of the HIV program, projected that two-thirds of the HIV response would be domestically funded by 2028 [30]. As an important step towards increased domestic contributions, SFI’s evidence generation and advocacy with the RGC and the GF helped to support successful negotiations to increase GF co-funding requirements for ARVs to $2.5 million per year for the 2021–2023 GF grant cycle, up from $1.5 million per year during the previous grant cycle [31]. The NAA went further, recommending a national budget allocation for ARVs of $3 million in 2021, $4 million in 2022, and $5 million in 2023 [32].

In 2019, Policy Circular 213 was ratified, expanding eligibility for the HEF to all PLHIV. Prior to the enactment of this new policy, only 38% of PLHIV had been eligible for the HEF, with less than half of those (18% of PLHIV) accessing services through the HEF. However, analyses supported by SFI indicated that an additional $428,000 in domestic resources would be needed to provide HIV services through the HEF to all poor PLHIV, and a five-fold increase in resources would be required to expand coverage to all PLHIV. These significant funding requirements meant that the expansion of HEF coverage under Policy Circular 213 would be implemented through a phased approach [29].

#### Outlook

Recognizing Cambodia’s progress in achieving epidemic control, domestic resource mobilization for HIV services to ensure the financial protection of PLHIV still remains an ongoing need. As Cambodia increases its commitments to fund treatment, additional funding will be needed for prevention services and to sustain civil society organizations that have played an important role in outreach to key populations. Operationalization of Policy Circular 213 is also still underway. Continued efforts to simplify and improve enrollment, data management, and payment processes for HEF; accelerate PLHIV enrollment into the scheme; and ensure sustainable financing for transportation reimbursements will be critical to ensuring long-term financial protection for PLHIV and sustained HIV epidemic control.

### Dominican Republic

#### Context

In recognition of high out-of-pocket payments, inequities, and quality and efficiency challenges within its health system, the DR passed a health reform law in 2001. As part of this reform, the *Seguro Familiar de Salud* (Family Health Insurance - SFS) was established. With both contributory and subsidized schemes, SFS was created with a mandate to provide universal coverage. Subsequently, the *Seguro Nacional de Salud* (National Health Insurance Agency - SENASA) was created to manage the subsidized scheme and contract health providers. With these structures in place, the DR reached 70% health insurance coverage by 2016 [33]. However, only an estimated 41% of PLHIV were enrolled in public health insurance [34].

The DR has made similar strides in its HIV response. With a concentrated HIV epidemic, the country has an HIV prevalence of 0.9% and 75,000 PLHIV [15]. There are 25,410 PLHIV of Haitian descent living in the DR, representing 34% of all PLHIV [35]. Since 2015, the Government of the DR (GoDR) has taken full responsibility for the costs of purchasing ARVs to meet the country’s needs [36]. The Ministry of Health has updated administrative and clinical care guidelines to reflect global best practices, and it has supported progress toward National Health Insurance coverage of ARVs as essential medicines. In 2019, the GoDR funded 61% of the country’s HIV response, with donor contributions accounting for 39% [20].

#### Activities

In collaboration with the GoDR and the non-governmental organization (NGO) sector, SFI supported a comprehensive, multi-year strategy aimed at increasing the sustainability of the HIV response and decreasing the financing gap. SFI’s assistance consisted of generating evidence for estimating health insurance financing requirements, supporting health insurance policy reforms, developing mechanisms to integrate the financing and procurement of ARVs into SENASA, and increasing Ministry of Health organizational capacity for cost-effective purchasing and warehousing of ARVs.

SFI also conducted a qualitative study to assess barriers to PLHIV enrollment in health insurance. Subsequently, SFI supported governmental and non-governmental organizations, including the National HIV and AIDS Council, to increase enrollment through training of staff at HIV clinics across the country, providing informational brochures regarding the benefits of enrolling into the national health insurance plan and the steps to do so, and development of guidelines to support the enrollment process. Moreover, since NGOs serve as critical HIV service providers for key and vulnerable populations in the DR, SFI worked to strengthen their capacity to meet national health insurance program requirements for accreditation.

#### Results

Working with the GoDR, SFI supported the mobilization of domestic resources and integration of financing, procurement, and distribution of ARVs into the country’s national health insurance program. SFI’s analyses identified efficiencies in ARV warehousing and distribution that were projected to save the government an estimated $160,000 annually [36].

As a result of SFI’s support for PLHIV enrollment, between December 2019 and December 2020, 6,700 PLHIV were newly enrolled in SENASA’s subsidized health insurance scheme. Moreover, of the five supported NGOs, three NGOs successfully contracted with SENASA and adopted new standard operating procedures to ensure efficient claims submissions and monitoring. As of May 2021, these three NGOs had received $111,300 in payments from SENASA. However, the two remaining NGOs decided against contracting with SENASA due to concerns about proposed reimbursement rates [34]. Where NGOs have successfully contracted with health insurance, they are now able to access new sources of funding while also facilitating expanded access to health services for their PLHIV clients.

#### Outlook

Strengthening HIV coverage through the national health insurance program supports both long-term coverage for clients and more sustainable financing for ARVs, while also enabling cost savings and more efficient procurement. The National HIV Strategic Plan 2021–2024 can further define the roles and responsibilities for government and other stakeholders, including NGOs and their contributions to HIV programming. Current challenges in the national health insurance scheme include ensuring adequate funding for ARVs to match coverage needs, advancing prevention and treatment optimization practices, and addressing reimbursements to NGOs that are below cost, which create a disincentive for continued HIV service provision.

### Nigeria

#### Context

Nigeria’s National Health Insurance Scheme (NHIS) was established in 1999 by the Federal Government of Nigeria with the goal of providing high quality, affordable, and accessible health care for all Nigerians [37]. However, access to health care remained extremely low; by 2016, only 4.2% of Nigerians, primarily from the formal sector, had enrolled in NHIS [38]. Moreover, budget allocation for health was trending in the wrong direction, decreasing from 7.2% of the national budget in 2014 to 5.8% by 2016 [39].

#### Activities

Within this challenging context, SFI identified an opportunity in 2016 as momentum around UHC was growing, facilitating a more favorable political climate for discussion around financial protection and integration of HIV. SFI built upon ongoing USAID support for UHC and began advocating for the inclusion of HIV within the national insurance scheme. Initially, this conversation was a non-starter due to the government’s belief that it would not be financially feasible to include HIV. In response, SFI supported actuarial studies in Lagos, Rivers, and Cross River States. The results of the Lagos State study showed that adding HIV services to the benefits package would increase annual premiums by the equivalent of only 60 cents per person [40].

By 2017, with evidence from the actuarial study showing that the cost of integrating HIV (including ARVs) would represent less than 5% of the total premium, the Government of Nigeria decided to move forward with HIV integration. Following this decision and given the decentralization of NHIS, which focuses implementation at state level with the NHIS providing guidance and regulation, SFI supported the development of a National HIV and Tuberculosis Blueprint in 2019. In May 2020, the NHIS formally adopted the blueprint, which serves as a guide on how to integrate both HIV and tuberculosis services into state health insurance schemes. As part of its advocacy efforts, SFI also supported the establishment of the Legislative Network for Universal Healthcare, which aims to leverage legislative functions in support of UHC and encourage effective collaboration between legislators and state ministries of health [40].

Seeking to support implementation for a proof of concept, SFI made the decision to focus on two states: Lagos and Kano. However, due to funding limitations and COVID-19-related challenges in Kano, by mid-2020, SFI decided to concentrate efforts in Lagos State. As the largest economy and a progressive center within Nigeria, Lagos State represented the best locus to gain initial success. To support the rollout of HIV benefits (limited to provider-initiated HIV testing at the outset) under the Lagos State Health Scheme, SFI supported the development of a roadmap for integrating HIV. Subsequently, SFI supported the design of the benefits package and referral system, enrollment of PLHIV, and empanelment of health facilities. SFI also facilitated meetings between the Lagos State Health Management Agency (LASHMA) and HIV commodity distributors in order to facilitate the purchase of HIV test kits through the empaneled facilities’ capitation funds.

#### Results

By leveraging Nigeria’s decentralized health system and catalyzing state-level integration of HIV into health insurance schemes, SFI’s support for the National HIV and Tuberculosis Blueprint proved to be a critical intervention. Engagement with legislators was also successful; within one year, five state legislatures enacted state health insurance scheme laws, and five states also increased their budget allocations for HIV.

In February 2022, LASHMA launched an expanded benefits package, which covers additional services including client-initiated HIV testing and HIV service charges (i.e., consultation, drug refill, and counseling fees). As of May 2022, 611,578 people were enrolled in the Lagos State Health Scheme, up from 11,000 at baseline (September 2020). Two hundred sixteen health facilities were empanelled under LASHMA, and 197 of those facilities are providing HIV testing services [41].

#### Outlook

In June 2021, the Government of Nigeria, through the National Agency for the Control of AIDS, launched a new National Domestic Resource Mobilization and Sustainability Strategy for HIV (2021–2025), which has the potential of mobilizing $662 million in domestic financing for HIV [42]. It includes goals to integrate comprehensive HIV services into the benefits packages of all state health insurance schemes by 2025 and to integrate comprehensive HIV services into major private health insurance schemes by 2023 [42]. The strategy represents an opportunity to continue expanding health insurance within Nigeria; however, concerted efforts to increase public financing to support the enrollment of vulnerable populations while also promoting increased informal sector participation will be critical for the viability of health insurance in Nigeria. Efforts in Lagos State continue to focus on expanding enrollment–including through targeted outreach to informal workers–with the plan to further expand the package of HIV services supported once enrollment surpasses two million.

### Cameroon

#### Context

Historically, out-of-pocket costs for health have been extremely high in Cameroon, reaching approximately 70% in 2012 and ranking Cameroon the third highest in sub-Saharan Africa [43]. Nearly two-thirds of households were unable to access health services due to financial barriers [43]. Although out-of-pocket spending for HIV is substantially lower, at just 6% due to high amounts of donor funding for HIV [18], financial barriers to accessing HIV services were still a concern.

In recognition of these challenges, Cameroon’s Health Sector Strategy (2016–2027) includes UHC as one of the key health financing reforms to be instituted [44]. In addition, PEPFAR has worked with the Government of the Republic of Cameroon (GRC) to eliminate user fees for HIV and related services, resulting in a new policy that took effect on January 1, 2020. Acknowledging the resultant funding gap, the government intends to ensure sustained funding for HIV through the incorporation of HIV services into UHC. As part of these efforts, in 2020, the GRC signed a deal with a South Korean company, entering into a public-private partnership agreement named *Santé Universelle Cameroun* (Universal Health Cameroon - SUCAM) to manage UHC for a period of 17 years [45]. SUCAM will be responsible for managing all commercial and financial operations for UHC implementation [46].

#### Activities

In Cameroon, SFI provided support directly in response to the government’s needs. The government, led by its UHC technical working group, developed a roadmap to guide the rollout of UHC, including a first phase focused on an expanded package of free or subsidized care for pregnant women, children under five, and PLHIV and a second phase to include broader coverage for the rest of the population. The government’s technical and financial partners, including USAID, through SFI and PEPFAR support, then stepped in to support specific components of that roadmap.

SFI focused its support on essential elements that would facilitate the rollout and implementation of UHC, while providing an opportunity to ensure that HIV considerations were appropriately integrated as systems were developed. Specifically, SFI provided technical assistance for the development of a UHC investment case, a consolidated package of interventions to be included as free services under UHC, standardized care and service protocols, and health facility UHC accreditation tools. In addition, SFI supported the Ministry of Health in updating its strategic communications plan for UHC; coordinating and delivering training on UHC to decentralized health and communications officials and health facility staff; and developing a range of communications materials to sensitize the general public about the upcoming rollout of UHC.

#### Results

Although the official launch of Cameroon’s UHC scheme is still pending the enactment of the health insurance law (originally expected in November 2021), SFI support has been instrumental in helping the GRC to achieve stepwise progress on key building blocks of the new system. SFI’s support helped to ensure that HIV services including HIV testing, treatment, and associated lab services, as well as condom distribution, were included in the consolidated package of services, and that the standardized care and service protocols and accreditation criteria both reflect the latest technical guidance for HIV. Moreover, the communications materials supported through SFI have been utilized by the government in prominent ways to advance the rollout of UHC; for example, SFI-produced materials were used at a June 2021 National Assembly meeting to advocate for the legal framework of UHC.

#### Outlook

By ensuring HIV services are fully integrated into UHC at the outset, SFI’s investment has helped to forge a path for long-term sustainability of the HIV response in Cameroon. However, sustainability is dependent on the success of UHC rollout and financing. The SUCAM partnership has introduced significant unknowns for development partners who have been working with the government on UHC rollout, as specifics around the agreement and SUCAM’s role have not been made publicly available. Continued engagement in the UHC rollout is needed to ensure that access to HIV services, particularly for the most vulnerable, remains a high priority as these broader systems are operationalized. And it is clear that government financing for health will need to increase substantially for UHC rollout to be feasible. The GRC has committed to mobilizing resources to sustainably finance UHC; ensuring that the government fulfills those commitments remains critical to achieving Cameroon’s UHC goals in the short to medium term, especially given the significant fiscal challenges arising from the COVID-19 pandemic.

## Lessons learned and recommendations

SFI generated a number of key lessons, focused around both how to successfully integrate HIV into financial protection systems, as well as what makes such systems effective.

### Understanding the local context and aligning interventions to local priorities–all under the leadership of local stakeholders–is paramount for achieving an effective and sustainable response

Government vision and leadership is critical for effective, sustainable systems-level interventions, especially given the substantial policy requirements required to roll out and change financial protection systems. Government leadership is also unique in its ability to bring all stakeholders together around a common objective. In Cambodia, such leadership was key, as numerous stakeholders including the Ministry of Health, National AIDS Authority, and National Center for HIV/AIDS Dermatology and STD—as well as partner organizations such as UNAIDS and the GF—all collaborated on the development of a guiding framework for integration of HIV/AIDS services into the existing health system and inclusion of these goals in the 10-year health sector plan. In addition, it is important to recognize that there are many contextual factors that will ultimately affect the speed and success of activities, as demonstrated by the differing levels of achievement among SFI’s financial protection interventions across nine countries. Program planners should consider timing, political climate, and systems readiness before introducing new activities, and programs must remain flexible enough to adapt to these contextual factors over time.

### There are many essential and mutually reinforcing components of expanding financial protection for PLHIV, and all of these must be considered

Policy work–such as Vietnam’s master plan for UHC–is an important foundation for financial protection systems. At the same time, monitoring of implementation to identify and address challenges and bottlenecks is also important. And while the design of financial protection systems is critical, financing of those systems is also a key consideration which must be considered from the start of planning; ultimately, increased domestic investment (discussed further elsewhere in this supplement [13]) is essential for achieving sustainability. Demand side interventions, such as the communications work implemented in Cameroon, are also important for ensuring strong uptake of new systems.

### When introducing large-scale systems reforms, a stepwise approach can help achieve success

For example, in Vietnam, HIV treatment services were initially integrated into SHI, with more recent efforts shifting to integration of prevention services. Similarly, in Nigeria, efforts were focused on Lagos state when national-level rollout of health insurance stalled. By focusing on smaller, manageable steps, these programs were able to demonstrate early results, which have helped to secure greater buy-in and funding, both from donors and domestic sources, for subsequent expansion.

### Equity is a critical consideration when building systems

Small decisions at the systems level can have outsized impacts–either positive or negative–on access to services for vulnerable populations, including key populations, orphans and vulnerable children, and those with low socioeconomic status. When designing financial protection systems, strategies such as adjusting enrollment criteria or subsidizing premiums should be considered in order to increase accessibility for these groups–precisely those who will benefit most from financial protection. Cambodia’s policy circular that expanded eligibility for the HEF to all PLHIV and Vietnam’s subsidization of premiums and ARV co-payments serve as two models for how equity considerations can be operationalized.

### Systems-level interventions require sustained investment over multiple years, but they have the potential to yield transformational impacts

It can take a significant amount of time–sometimes several years–to achieve sufficient momentum and start seeing measurable impacts. However, investments in financial protection systems can have profound and long-lasting impacts throughout the health system. In particular, in countries where HIV services have developed as an emergency response through parallel systems, integration of HIV into financial protection systems can provide a platform for overall integration of those parallel systems, creating efficiencies and better quality of care for clients. This was the case in Vietnam, where the integration of HIV treatment services into SHI helped to spur both efficiencies and increased domestic financing for HIV, and where continued access to treatment for PLHIV is now primed to continue uninterrupted as donor funding declines. We should continue to look for opportunities to drive such transformational changes, including through the alignment of HIV investments with broader momentum around UHC.

### Although financial returns may not be the most important measure of success, investments in financial protection systems can produce positive returns in the medium term

Over a period of six years, PEPFAR achieved a return of $2.22 for every dollar invested in Vietnam’s SHI program. The significant investments that PEPFAR made to support policy and legal changes are projected to produce even greater returns in the future since outcomes have been institutionalized by the GVN. However, where policy work and operationalization of financial protection systems have been prolonged (Cambodia and Cameroon) or where enrollment and empanelment of health facilities are still in the initial scale-up phase (DR and Nigeria), it may not be feasible to understand PEPFAR’s return on investment for several years. However, once these systems are fully operationalized and a critical mass of clients is enrolled, positive returns are expected.

## Conclusions

As more and more countries reach epidemic control, and donor funding for HIV continues to decline, sustainable financing for HIV services is becoming a growing priority. Although the ultimate impact of SFI’s investments in financial protection has not yet been fully realized, results to date demonstrate that with strong political will; long-term engagement with partner governments; and focused technical assistance for advocacy, policy reform, and implementation support, HIV services can be successfully integrated into financial protection schemes. Such integration can promote increased, long-term domestic financing for HIV while also protecting PLHIV from financial risk.

SFI’s experience has also shown that successfully institutionalizing new systems requires considerable effort over many years, and therefore the time to start these interventions is now. Moreover, as the global community faces myriad health and economic challenges brought about by COVID-19, the need for resilient health systems that can effectively respond to shocks has become increasingly clear. There is a great opportunity for donors, including PEPFAR and GF, to join forces with partner governments and their ongoing UHC efforts to invest in financial protection systems and support the achievement of broader goals of equity, access to health, and resilience.

## Data Availability

Data and materials used and/or analyzed for this manuscript are available from the corresponding author on reasonable request.

## Abbreviations

ARV: Antiretroviral drug
DR: Dominican Republic
GF: The Global Fund to Fight AIDS, Tuberculosis and Malaria
GoDR: Government of the Dominican Republic
GRC: Government of the Republic of Cameroon
GVN: Government of Vietnam
LASHMA: Lagos State Health Management Agency
NGO: Non-governmental organization
NHIS: National Health Insurance Scheme (Nigeria)
PEPFAR: U.S. President’s Emergency Plan for AIDS Relief
PLHIV: People living with HIV/AIDS
RGC: Royal Government of Cambodia
SENASA: Seguro Nacional de Salud (National Health Insurance Agency-DR)
SFI: Sustainable Financing Initiative
SFS: Seguro Familiar de Salud (Family Health Insurance-DR)
SHI: Social Health Insurance (Vietnam)
SUCAM: Santé Universelle Cameroun (Universal Health Cameroon)
UHC: Universal health coverage
UNAIDS: The Joint United Nations Programme on HIV/AIDS
USAID: U.S. Agency for International Development
VAAC: Vietnam Administration for AIDS Control
